# Casticin inhibits AKR1C3 and enhances abiraterone efficacy in castration-resistant prostate cancer

**DOI:** 10.1007/s11418-025-01974-8

**Published:** 2025-12-10

**Authors:** Kamil Piska, Michał Zubek, Adam Bucki, Maria Świtalska, Paulina Koczurkiewicz-Adamczyk, Benedykt Władyka, Marcin Kołaczkowski, Elżbieta Pękala

**Affiliations:** 1 Department of Pharmaceutical Biochemistry, Faculty of Pharmacy, Jagiellonian University Medical College, Medyczna 9 St, Kraków, Poland; 2 Department of Medicinal Chemistry, Faculty of Pharmacy, Jagiellonian University Medical College, Kraków, Poland; 3 Department of Analytical Biochemistry, Faculty of Biochemistry, Biophysics and Biotechnology, Jagiellonian University, Kraków, Poland

**Keywords:** Vitexicarpin, 17β-hydroxysteroid dehydrogenase type 5, Steroids, Prostate cancer, Ethnopharmacology

## Abstract

Castration-resistant prostate cancer (CRPC) continues to represent a critical therapeutic hurdle owing to its resistance to both androgen deprivation and next-generation antiandrogens like abiraterone (ABI). One of the key mechanisms underlying this resistance involves overexpression of aldo-keto reductase 1C3 (AKR1C3), an enzyme contributing to intratumoral androgen biosynthesis. In this study, casticin (CAS), a flavonoid derived from *Vitex agnus-castus*, was identified as a potent inhibitor of AKR1C3. CAS showed potent inhibitory activity in enzymatic assays (IC₅₀ = 5.99 µM), significantly suppressed AKR1C3-mediated coumberone metabolism in 22Rv1 prostate cancer cells, and showed greater cytotoxicity in AKR1C3-expressing 22Rv1 cells relative to AKR1C3-deficient LNCaP cells. CAS significantly enhanced ABI’s cytotoxic efficacy in 22Rv1 cells, as evidenced by synergistic interactions (CI: 0.31–0.71); however, no such synergy was observed in LNCaP cells or with enzalutamide. CAS enhanced apoptosis in ABI-treated 22Rv1 cells, as well as combination showed only a limited effect against normal epithelial PNT-2 cell line. Docking and molecular dynamics simulations indicated a stable CAS–AKR1C3 interaction, characterized by crucial hydrogen bonding and aromatic stacking within the active site. These results suggest that CAS is a promising chemosensitizer targeting AKR1C3 to overcome ABI resistance in CRPC.

**Graphical Abstract:**

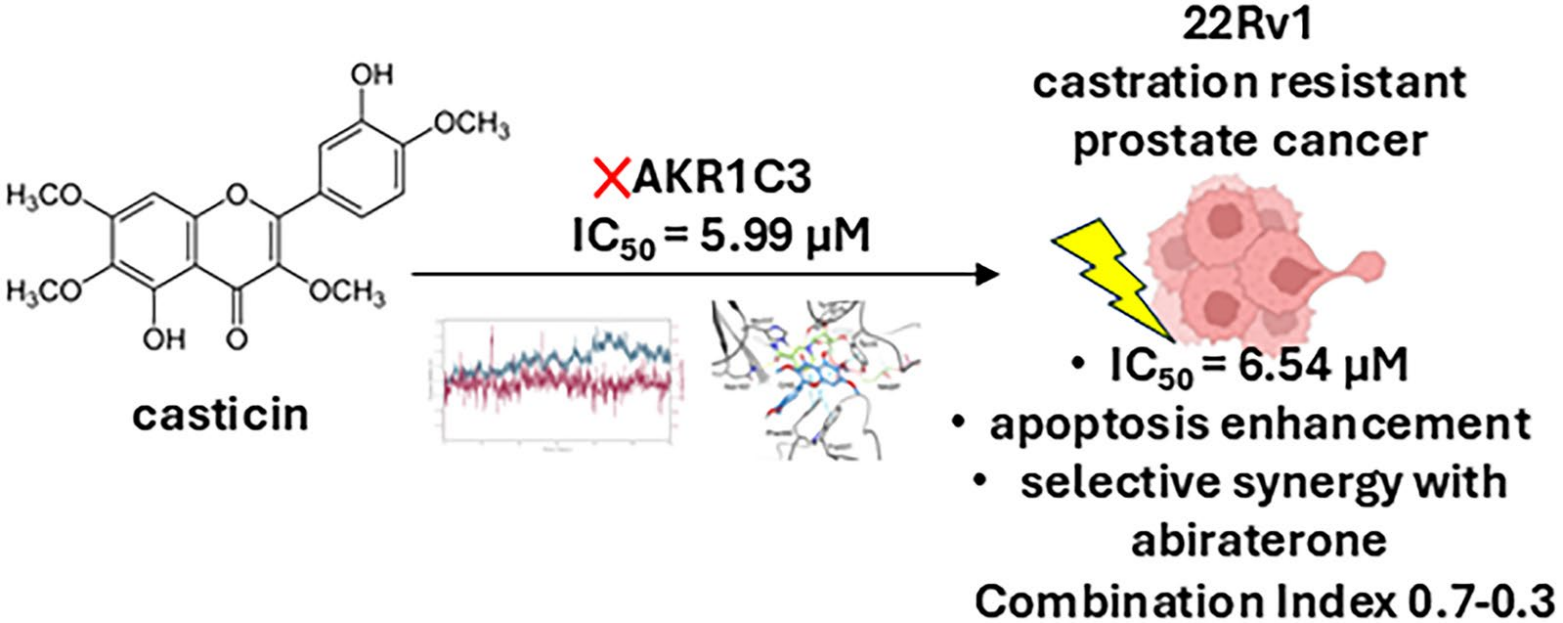

**Supplementary Information:**

The online version contains supplementary material available at 10.1007/s11418-025-01974-8.

## Introduction

Prostate cancer is one of the most prevalent malignancies in men worldwide and represents a growing public health concern, especially in developed countries. It accounts for a substantial proportion of cancer diagnoses and mortality, with rising incidence attributed to population aging and advancements in diagnostic methods. Despite considerable progress in screening, early detection, and therapeutic approaches, prostate cancer remains a leading cause of cancer-related morbidity and mortality in men [1].

A major challenge in prostate cancer management is the development of resistance to conventional treatments. Although androgen deprivation therapy is initially effective in most patients with advanced disease, tumors frequently progress to a more aggressive and lethal form known as castration-resistant prostate cancer (CRPC) [2]. In recent years, next-generation antiandrogens such as enzalutamide (ENZ) and androgen biosynthesis inhibitors like abiraterone (ABI) have improved clinical outcomes in patients. However, resistance to these agents commonly arises, significantly limiting their long-term efficacy and emphasizing the urgent need for alternative therapeutic strategies [3].

Aldo-keto reductase 1C3 (AKR1C3; 17β-hydroxysteroid dehydrogenase type 5) has emerged as a key molecular target in therapy resistance. This enzyme plays a critical role in intratumoral androgen biosynthesis by catalyzing the NADPH-dependent reduction of 4-androstene-3,17-dione (androstenedione) to testosterone and of 5α-androstane-3,17-dione to dihydrotestosterone (DHT). These reactions represent critical steps in the intracrine production of potent androgens that maintain androgen receptor (AR) signalling in CRPC, even under systemic androgen deprivation [4, 5]. Additionally, AKR1C3 acts as an AR coactivator and stabilizes AR by preventing its ubiquitination [6, 7]. Elevated AKR1C3 expression is linked to poor prognosis in prostate cancer and correlates with androgen receptor splice variants (e.g., AR-V7), which exhibit ligand-independent activity [7–9]. As a promising drug target to overcome resistance, inhibitors of this enzyme are being sought from natural sources and through rational drug design. ASP9521 was tested in clinical trials for prostate cancer, and currently, indomethacine (IND) for a novel AKR1C3 inhibitor is under evaluation [4, 10]. Interestingly, several natural flavonoids have been identified as AKR1C3 inhibitors, including genistein, liquiritigenin, and 2′-hydroxyflavanone [11–13].

Casticin (CAS), a major flavonoid derived from Chaste tree (*Vitex agnus-castus* L.; VAC) fruits, has demonstrated promising anticancer activity in several types of malignancies, including breast, lung, and prostate. Its ability to inhibit cell proliferation, induce apoptosis, and interfere with key oncogenic signalling pathways makes it a candidate of interest in the search for novel treatments [14]. Interestingly, although VAC is commonly used to treat premenstrual syndrome, mastalgia, and hyperprolactinemia, it has historically been regarded as an antiandrogenic agent.

Traditionally, *Vitex agnus-castus* (VAC) was used in ancient Rome as an anaphrodisiac, with seed and leaf preparations believed to suppress sexual desire. In Christian monasteries, it earned the name “monks’ pepper” due to its perceived role in supporting celibacy. Classical authors such as Pliny the Elder, Dioscorides, and Theophrastus associated the plant with chastity and infertility. Its Latin name, *agnus castus*, reflects this symbolic and practical association with sexual abstinence [15]. There are also some modern studies suggesting the potential of VAC in decreasing the serum level of testosterone [16, 17].

Based on the significant role of CAS in the pharmacological activity of VAC and historical reports of the plant’s antiandrogenic effects, CAS - being a flavonoid constituent - was investigated as a potential inhibitor of AKR1C3. Given the enzyme’s established role in resistance to current androgen-targeting therapies, identifying novel AKR1C3 inhibitors is of particular therapeutic relevance [10].

Unlike previously reported natural AKR1C3 inhibitors such as genistein or liquiritigenin, which primarily act as enzymatic modulators without cytotoxicity, CAS uniquely combines AKR1C3 inhibition with potent, direct cytotoxic and pro-apoptotic activity. These support its further investigation in the context of treatment-resistant prostate cancer [14]. Additionally, CAS exhibits favourable physicochemical and pharmacokinetic properties, including oral bioavailability in rats, further supporting its potential as a drug candidate [18].

CAS was evaluated as an inhibitor in a kinetic assay using recombinant AKR1C3 and was found to inhibit AKR1C3-dependent coumberone reduction in cellular assay. CAS was also found to reverse resistance to ABI in castration resistant 22Rv1 AKR1C3-positive prostate cancer cell line, in contrast to LNCaP prostate cancer cell line which lacks AKR1C3 expression. The basis of CAS interactions with AKR1C3 was investigated using molecular docking and molecular dynamics simulations.

## Materials and methods

### Chemicals

CAS (98% purity) and ENZ (98%) were from Cayman Chemicals. ABI acetate (99%) was from Thermo Scientific Chemicals. 9,10-Phenanthrenequinone (95%), indomethacin (98%) were from Sigma Aldrich. Coumberone (99%) and ASP9521 (98%) were from MedChem Express. The tested compounds were initially dissolved in DMSO (Sigma-Aldrich) at a concentration of 25 mM and subsequently diluted in appropriate buffers and media. Corresponding DMSO vehicle controls were included in all experiments.

### Recombinant AKR1C3

Recombinant AKR1C3 protein was expressed in *Escherichia coli* Rosetta (DE3) transformed with pET21b carrying an E. coli-codon-optimized sequence coding for hAKR1C3 introduced between NdeI/XhoI restriction sites (GenScript). Cells were grown in LB medium supplemented with ampicillin (100 µg/ml) at 37 °C till the optical density at 600 nm reached the value of 0.6–0.8, then temperature was lowered to 22 °C and the expression of recombinant protein was induced by IPTG (final concentration 1 mM), and the cells were cultivated for further 4 h. Then, bacteria were harvested by centrifugation (10 000 g, 15 min), resuspended in Lysis buffer (50 mM Tris-HCl, pH 8.0, 300 mM NaCl, 10 mM imidazole), and sonicated. The lysate was clarified by centrifugation (21000 g, 20 min) and loaded on NiNTA resin. Subsequently, the column was washed with Lysis buffer, and AKR1C3 was eluted with Elution buffer (50 mM Tris-HCl, pH 8.0, 300 mM NaCl, 500 mM imidazole). The protein was further purified by gel filtration in 20 mM Tris-HCl, pH 7.4, 50 mM NaCl, 1 mM DTT, 1 mM EDTA using HiLoad 26/600 Superdex 200 pg. The procedure resulted in a pure preparation of AKR1C3, as evidenced by SDS-PAGE.

### AKR1C3 Inhibition

A reaction mixture contained 0.1 M phosphate buffer (pH 7.4), recombinant AKR1C3 enzyme (0.2 µM), substrate (9,10-Phenanthrenequinone, 10 µM), and CAS or reference inhibitor in a concentration range. 200 µM NADPH was added to initiate the reactions, and NADPH oxidation process was determined by measuring the decrease in absorbance at 340 nm (SpectraMax^®^ iD3, Molecular Devices) over 10 min. Reaction velocity was determined using linear regression in OriginPro, and the percentage decrease was calculated relative to the vehicle-treated control. DMSO at used concentration (0.7%) did not influence the enzyme activity. No enzyme-independent NADPH/substrate reaction was observed.

### Cell culture

22Rv1 (ATCC, CRL-2505) and LNCaP (ECACC, CRL 1740) prostate cancer cell lines and PNT-2 (Sigma–Aldrich cat. No. 95012613) prostate epithelial cell lines were cultured in standard conditions (37 °C, 5% CO_2_) in phenol red-free RPMI 1640 medium (Gibco) supplemented with 10% FBS and 1% antibiotics (Gibco). PNT-2 cells were additionally supplemented with glutamine (2 mM). For experiments (viability, coumberone reduction, apoptosis) 10% FBS was replaced with 10% charcoal-treated FBS (Gibco). All cell lines were routinely tested for Mycoplasma contamination using MycoStrip (Invitrogen).

### Coumberone reduction assay

22Rv1 cells were seeded in 96-well plates at a density of 2 × 10⁴ cells per well and incubated overnight at 37 °C. The following day, cells were pretreated with CAS or reference compounds for 1 h, followed by the addition of coumberone at a final concentration of 10 µM. After 24 h of incubation, 100 µl of the culture medium was transferred to a black 96-well plate for fluorescence measurement. Fluorescence intensity was measured with an excitation wavelength of 385 nm and emission wavelength of 510 nm using a plate reader (Spectra Max iD3, Molecular Devices) and normalized to cell viability in SRB assay. Cells treated with coumberone alone were used as a control, representing 100% coumberone reduction. Data represent the mean of three independent experiments, each performed in triplicate.

### Sulforhodamine B viability assay

Cells were seeded in 96-well plates at a density of 1 × 10⁴ (22Rv1) or 5 × 10³ (LNCaP, PNT-2) cells per well. After 24 h, solutions of CAS, reference agents and vehicle were preincubated for 3 h, and next ABI, ENZ or vehicle were added. After 72 h of incubation, cells were fixed with 50% (w/v) trichloroacetic acid for 1 h at 4 °C. Cells were washed with water, and stained for 30 min with sulforhodamine B solution (0.4% in 1% acetic acid). Then cells were washed four times with 1% acetic acid, and the incorporated stain was solubilized in 10 mM Tris solution. Absorbance was measured at 565 nm using a SpectraMax iD3 microplate reader (Molecular Devices). Viability was calculated by dividing the average absorbance of each experimental condition by the absorbance of the vehicle control, multiplied by 100 (%). Data represent the mean of three independent experiments, each performed in triplicate.

### Combination index

To quantify drug interaction, the Combination Index (CI) was estimated by the Chou-Talalay method using CompuSyn software (ComboSyn, Inc. NY, USA).

### Caspase 3/7 activity

Investigation of caspases 3 and 7 activity in cells were assessed with Caspase-Glo^®^ 3/7 Assay System (Promega). 22Rv1 cells were seeded in 96-well white plates at a density of 1 × 10⁴ cells per well. After 24 h, solutions of CAS, reference agents and vehicle were preincubated for 3 h, and next ABI or vehicle were added. After 72 h assay was performed. Briefly, Caspase-Glo^®^ 3/7 Reagent was added to the each well and after 30 min luminescence was read using a SpectraMax iD3 microplate reader (Molecular Devices).

### Hoechst 33342 staining

Investigation of the morphology of cell nuclei as a marker of apoptosis was performed. 22Rv1 cells were seeded in 96-well white plates at a density of 2.5 × 10⁴ cells per well. After 24 h, solutions of CAS, reference agents and vehicle were preincubated for 3 h, and next ABI or vehicle were added. After 72 h assay was performed. Cells were treated with a medium containing Hoechst 33342 (Thermofisher Scientific) at concentration of 2 µM. After 10 min cells were washed with PBS and observed with Leica DMiL LED Fluo in a visible light and a fluorescence mode.

### Statistics

Statistical significance was determined using Student’s t-test, with p-values < 0.05 considered significant. For multiple comparisons, Bonferroni correction was applied. All analyses were performed using GraphPad Prism.

### Molecular modeling

The presented molecular modelling studies were performed using the Small-Molecule Drug Discovery Suite 2024-4 (Schrödinger Inc.), on a workstation operated by a Linux Ubuntu 24.04 LTS system. The CAS ligand was prepared by LigPrep and docked using the Induced-Fit Docking protocol to an initial AKR1C3 model, which was based on the 4DBS experimental structure [19], downloaded from the RCSB PDB and processed using the Protein Preparation Workflow. The conformational model reflecting the best-scored complex was used for re-docking in the Glide SP procedure (with a constrained H-bond to His117), and re-scoring using the MM-GBSA minimisation algorithm.

The final complex was directed to MD simulations carried out by the Desmond GPU software (D.E. Shaw Research and Schrödinger Inc.). First, it was relaxed using a Brownian motion simulation for 1 ns. The production phase was carried out for 120 ns, at a temperature of 310 K, and a pressure of 1.01325 bar (NPT ensemble class), with a trajectory and energy recording interval of 100 ps (1200 frames in total). Three independent replicates with different random seeds and initial velocities were performed. Following each run, the Simulation Interaction Diagram was used for RMSD plotting and generation of interaction timelines.

## Results

### Recombinant AKR1C3 Inhibition

**Fig. 1:**
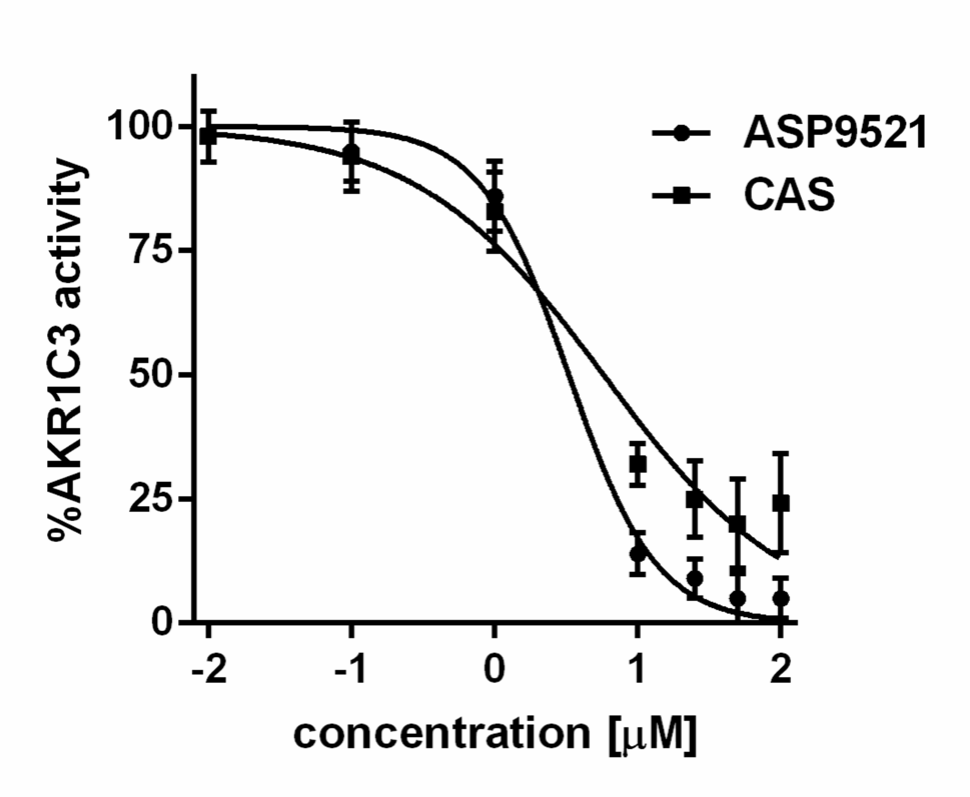
Activity of recombinant AKR1C3 with CAS and standard AKR1C3 inhibitor ASP9521. Data represent the mean ± SD of three independent experiments, each performed in triplicate

CAS inhibited AKR1C3 with potency comparable to that of the reference selective inhibitor ASP9521. The IC₅₀ value of CAS was 5.99 µM, compared to 3.32 µM for ASP9521 (Fig. 1).

### AKR1C3 Inhibition in 22Rv1 cells

**Fig. 2:**
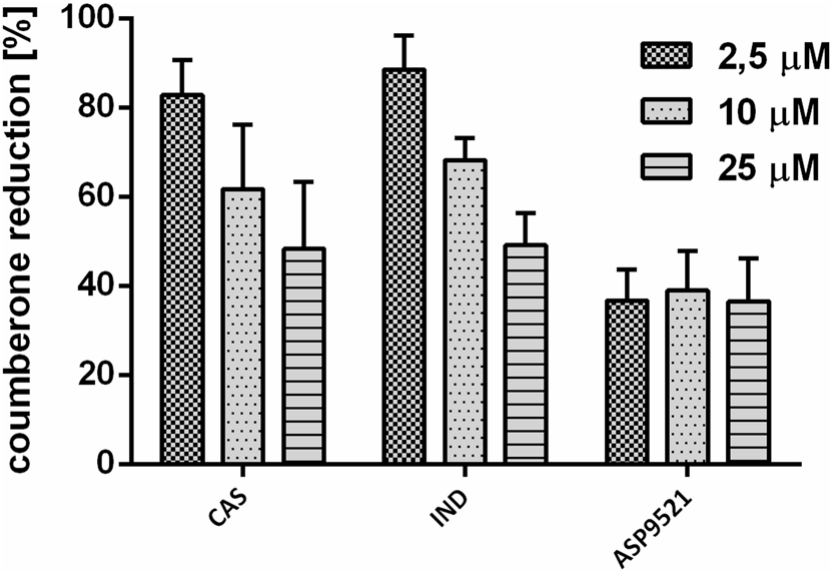
Coumberone reduction in 22Rv1 cells was decreased by CAS and reference AKR1C3 inhibitors (IND, ASP9521). 100% coumberone reduction is related to vehicle control. Data represent the mean ± SD of three independent experiments, each performed in triplicate

Coumberone is a substrate for AKR1C3-catalysed reaction, which results in the formation of coumberol. While the product exhibits fluorescence with excitation and emission wavelengths of 385 nm and 510 nm, respectively, the substrate does not. Therefore, the increase of the fluorescence of a medium with coumberone incubated with the cell culture is related to the reduction process. CAS inhibited coumberone reduction to a similar extent as indomethacin (IND). ASP9521 possessed much higher activity; it inhibited coumberone reduction in the highest percentage, even at the concentration of 2.5 µM (Fig. 2).

### Cytotoxicity assessment

**Fig. 3:**
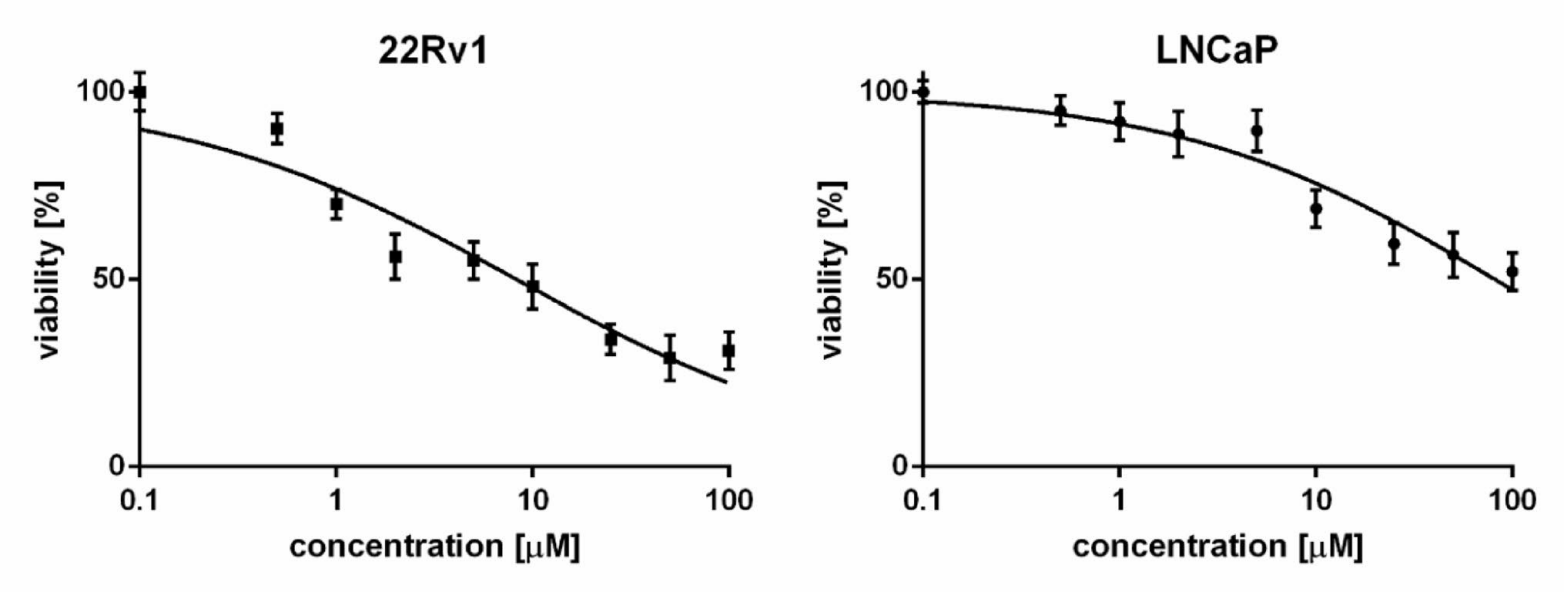
Viability of 22Rv1 (A) and LNCaP (B) cells incubated with CAS for 72 h in SRB assay. Data represent the mean ± SD of three independent experiments, each performed in triplicate

CAS was evaluated for its cytotoxic and chemosensitizing effects in 22Rv1 and LNCaP cell lines. While 22Rv1 cell line is characterized by high expression of AKR1C3, LNCaP cell line is known to present a lack or trace level of AKR1C3. Both cell lines express androgen receptor and are androgen-dependent. Therefore, comparison of different biological effects on these cell lines is used to estimate the contribution of AKR1C3 to the activity.

In the viability assay, CAS was incubated with 22Rv1 and LNCaP for 72 h, next SRB assay was performed. CAS exhibited cytotoxic activity in both cell lines (22Rv1 IC_50_ = 6.54 µM; LNCaP IC_50_ = 70.90 µM). However, its potency was markedly higher in 22Rv1 cells than in LNCaP cells (Fig. 3).

### Drug combinations

**Fig. 4:**
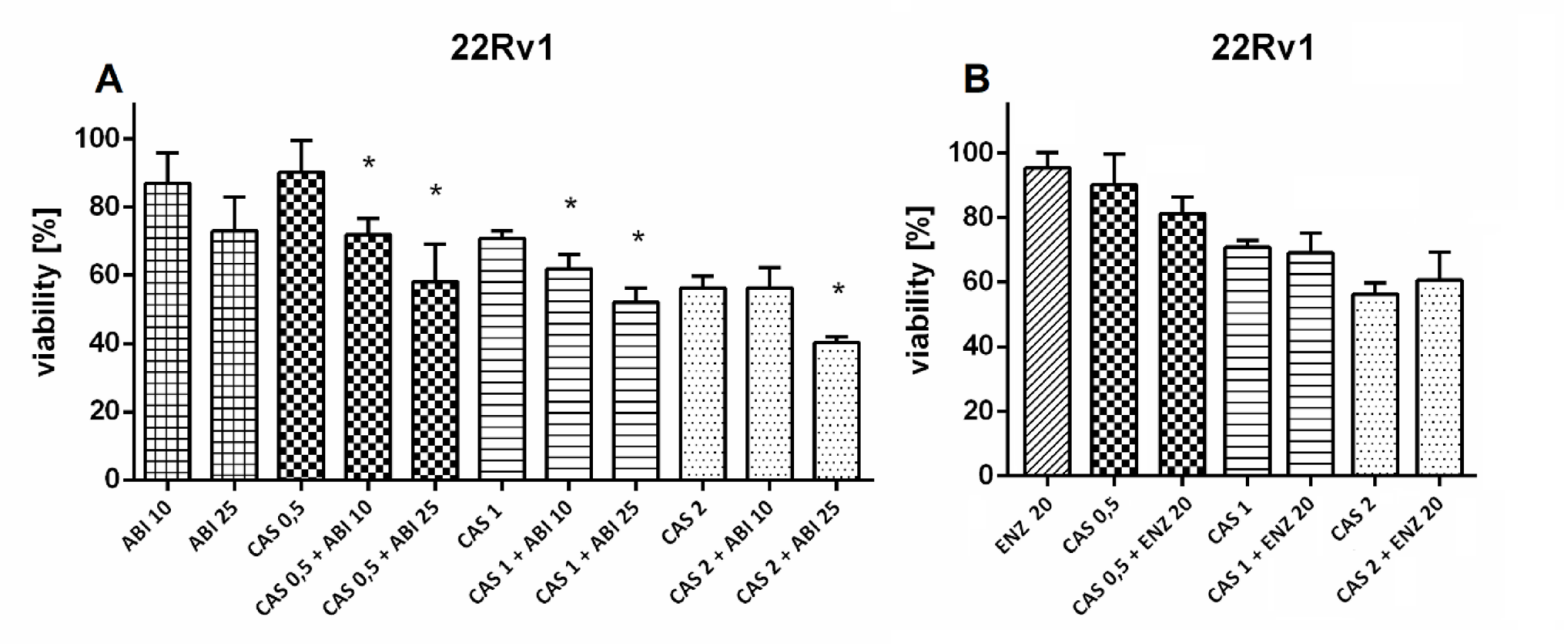
Drug combination assay in 22Rv1 cells. Cells were preincubated with CAS for 3 h, after which ABI or ENZ was added. After 72-hour incubation SRB assay was performed. Numbers next to drugs’ names represent µM-concentrations. Data represent the mean ± SD of three independent experiments, each performed in triplicate. * statistical significance (*p* < 0.05) is indicated when the combined treatment condition differs significantly from both CAS alone and ABI or ENZ alone (t-Student test with Bonferroni correction)

**Fig. 5:**
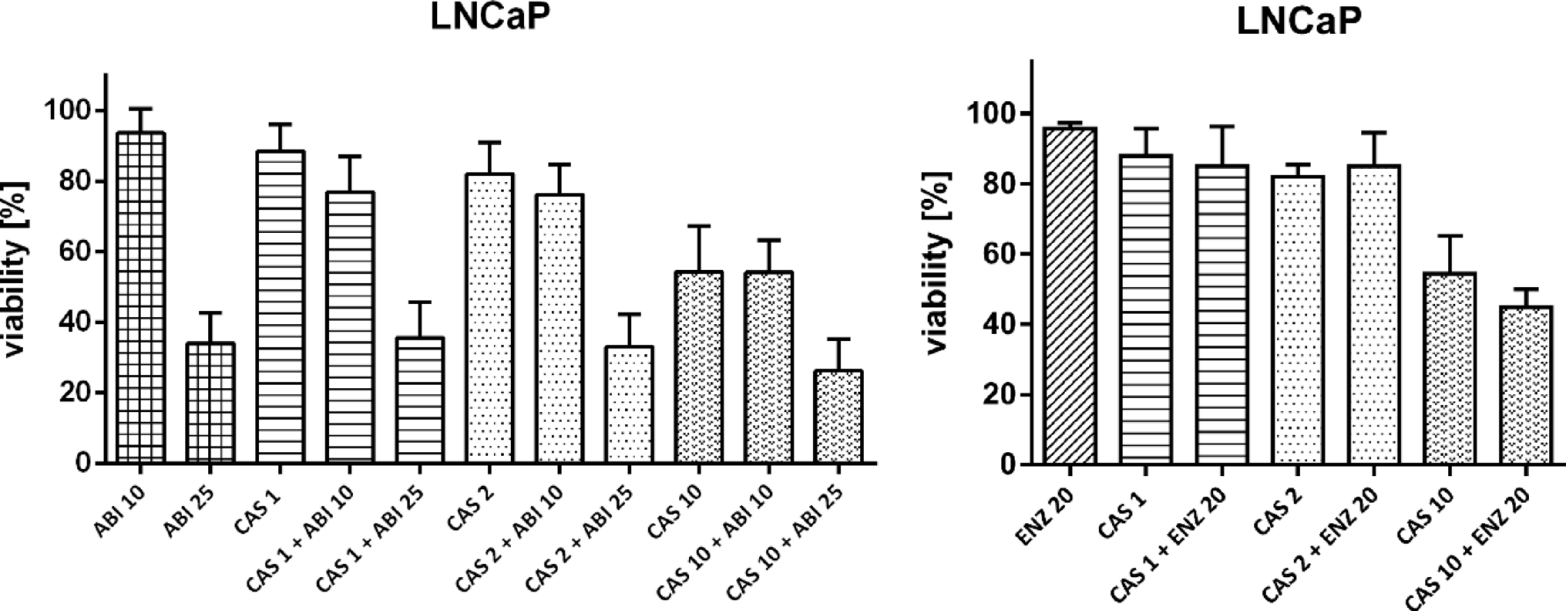
Drug combination assay in LNCaP cells. Cells were preincubated with CAS for 3 h, after which ABI or ENZ was added. After 72-hour incubation SRB assay was performed. Numbers next to drugs’ names represent µM-concentrations. Data represent the mean ± SD of three independent experiments, each performed in triplicate. No statistically significant (*p* < 0.05) differences between the combined treatment condition and CAS alone and ABI/ ENZ alone were found (t-Student test with Bonferroni correction)

CAS was tested in combination with ABI and ENZ, both of which are used in CRPC treatment. In 22Rv1 cell line, CAS in concentrations of 0.5, 1, and 2 µM was found to synergize with ABI in concentrations of 10 µM and 25 µM. In five out of six combinations, the reduction in cell viability was statistically significant compared to both CAS alone and ABI alone at the corresponding concentrations (Fig. 4A). Combination Index calculated by Chou-Talalay method was used to measure the potency of observed interactions. Its values range from 0.71 − 0.31, indicating a robust potency of a synergy (Table 1, Fig. S1).

No statistically significant reduction in viability was observed for the CAS and ABI combination compared to either drug alone in AKR1C3-low expressing LNCaP cells (Fig. 5A). This suggests that the observed effect in 22Rv1 cells may be mediated by AKR1C3 activity. No interaction was also found between CAS and ENZ in both cell lines (Figs. 4B and 5B).

**Table 1:** Combination index (CI) values for CAS and ABI in 22Rv1 cells. CI values < 0.3 indicate strong synergy; 0.3–0.7, robust; 0.7–0.85, moderate; and 0.85–0.9, slight synergy

CAS [µM]	ABI [µM]	CI
0.5	10	0.71
	25	0.59
1.0	10	0.49
	25	0.49
2.0	10	0.52
	25	0.31

### Apoptosis induction

**Fig. 6:**
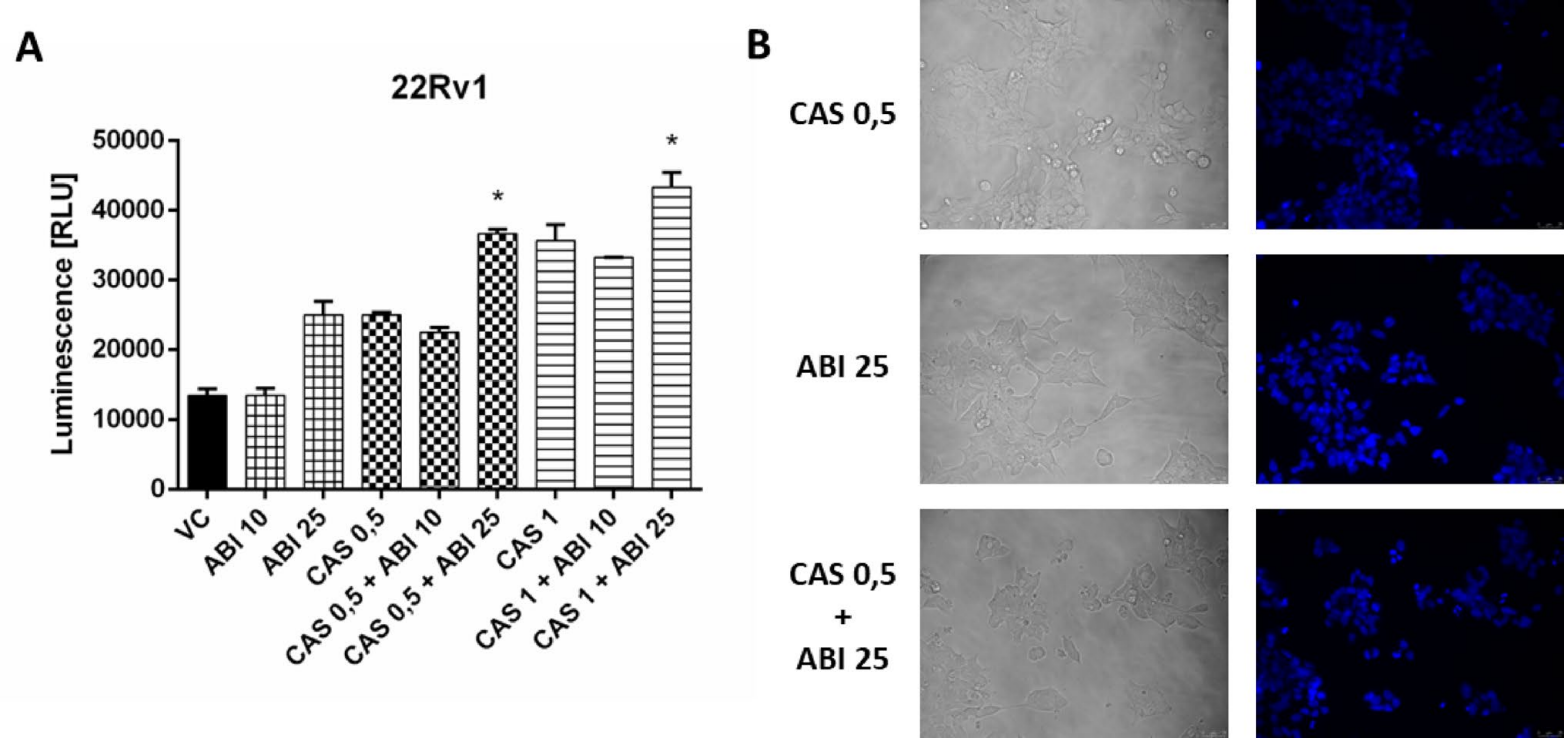
Evaluation of apoptosis following drug combination treatment. 22Rv1 cells were preincubated with CAS for 3 h, followed by the addition of ABI. After 72 h, caspase-3/7 activity was measured using a luminometric assay (**A**), and apoptotic bodies were assessed by Hoechst 33342 staining (20x magnification) (**B**). Numbers next to drugs’ names represent µM-concentrations. Data represent the mean ± SD of three independent experiments, each performed in triplicate. * statistical significance (*p* < 0.05) is indicated when the combined treatment condition differs significantly from both CAS alone and ABI or ENZ alone (t-Student test with Bonferroni correction)

To evaluate apoptosis induced by drug combinations, caspase-3 and −7 activity, as well as Hoechst 33342 staining of apoptotic bodies, were assessed in 22Rv1 cells (Fig. 6). Cells were treated in a manner analogous to the SRB assay, followed by the appropriate apoptosis assays. Both CAS and ABI were found to increase caspase-3/7 activity individually, with ABI at a concentration of 25 µM showing a notable effect. Importantly, combined treatment with CAS and ABI significantly enhanced caspase activity compared to either agent alone. The increase in apoptosis was further confirmed by Hoechst 33342 staining. While only a slight number of apoptotic bodies was observed in cells treated with CAS (0.5 µM) or ABI (25 µM) alone, a marked increase was observed following combined treatment. More representative images of Hoechst staining may be found at Supplementary Information.

### Drug combination in normal prostate cells

**Fig. 7:**
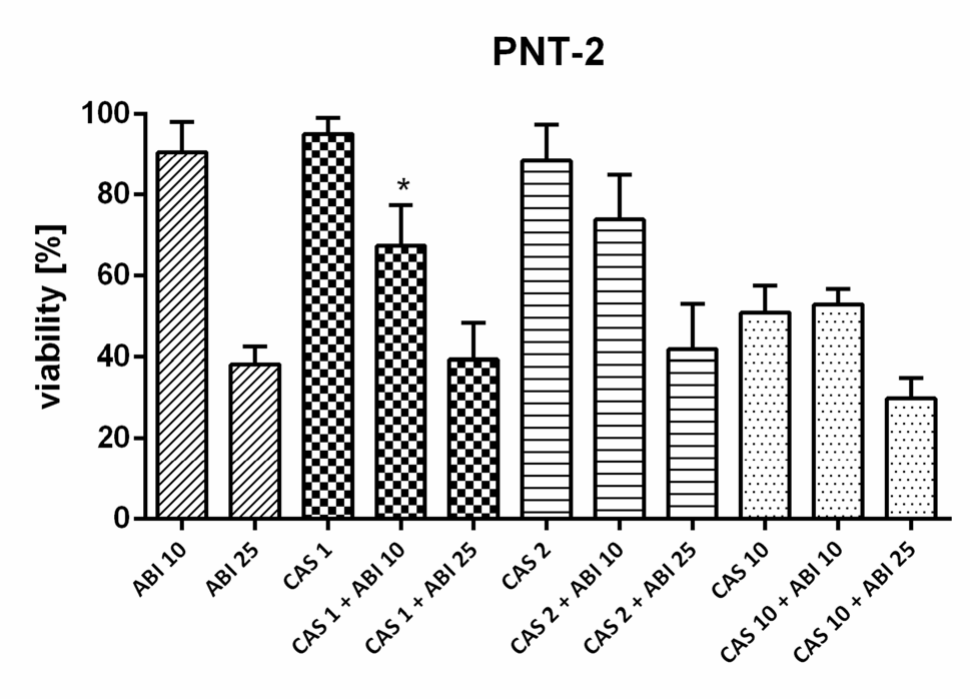
Drug combination assay in PNT-2 cells. Cells were preincubated with CAS for 3 h, after which ABI was added. After 72-hour incubation SRB assay was performed. Numbers next to drugs’ names represent µM-concentrations. Data represent the mean ± SD of three independent experiments, each performed in triplicate. * statistical significance (*p* < 0.05) is indicated when the combined treatment condition differs significantly from both CAS alone and ABI alone (t-Student test with Bonferroni correction)

In order to investigate safety of CAS and ABI combination against normal cells, PNT-2 epithelial prostate cell line was used (Fig. 7). While some weak trend to increase cytotoxicity after drug combination was noted, the change was statistically significant in only one combination, indicating lower significance than in 22Rv1 cells.

### Molecular docking

**Fig. 8:**
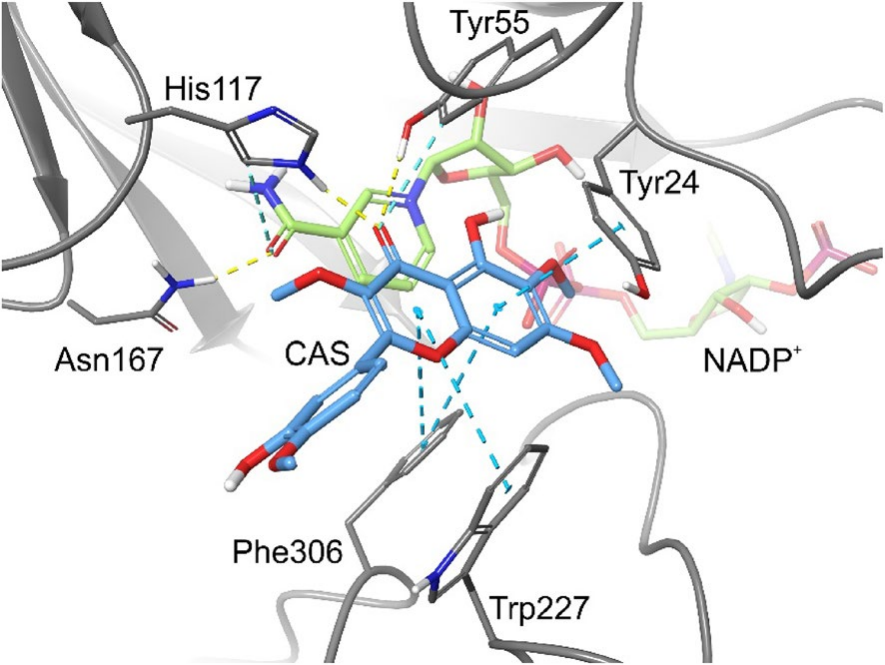
Predicted binding mode of CAS (blue) in the AKR1C3 active site. The ligand forms hydrogen bonds with His117 and Tyr55 (yellow dashed lines) and π-aromatic interactions with Tyr24, Trp227, and Phe306 (cyan dashed lines). The protein model is based on the experimental structure PDB ID: 4DBS, co-crystallised with NADP^+^ (pale green)

To elucidate the robust inhibitory activity of CAS against AKR1C3, molecular modelling tools were employed. CAS was docked into the structural model of AKR1C3, revealing a binding mode characteristic of known inhibitors within the orthosteric site, with a Glide gscore of − 9.84, and MM-GBSA score − 59.26 kcal/mol. The carbonyl group acted as a hydrogen bond acceptor, forming interactions with His117 and Tyr55, two key residues involved in catalysis. Additionally, the chromen-4-one ring was stabilised through extensive π-aromatic interactions with Tyr24, Trp227, and Phe306 (Fig. 8).

### Molecular dynamics

**Fig. 9:**
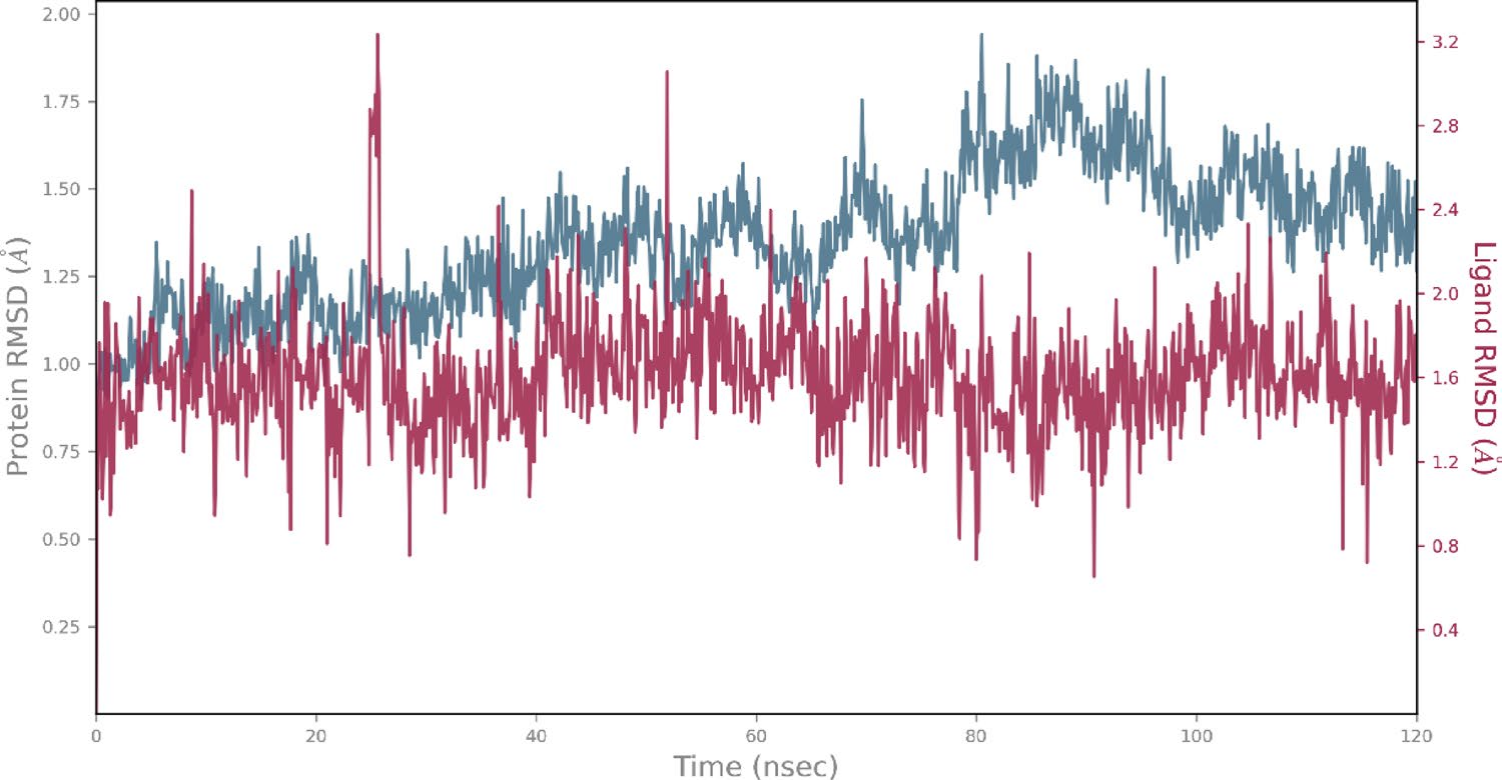
Protein and ligand RMSD values over the 120 ns MD simulation (replicate #1) of the CAS-AKR1C3 complex, indicating minimal conformational changes and sustained ligand residence

To evaluate interaction stability and active site residence time, molecular dynamics (MD) simulations were performed. The system, based on the docking-predicted CAS-AKR1C3 complex, retained all key interactions during the initial 1 ns minimisation step. Subsequent 120 ns MD simulations, performed in triplicate, produced stable trajectories with protein RMSD values below 2 Å and ligand RMSD values within 4.0 Å (for representative RMSD plot from simulation replicate #1 see Fig. 9).

The above interactions collectively contributed to the stable retention of CAS within the orthosteric binding site, supporting the in vitro findings and suggesting a favourable residence time consistent with potent AKR1C3 inhibition [20]. Results from replicates #2 and #3 are provided in the Supplementary Information.

**Fig. 10:**
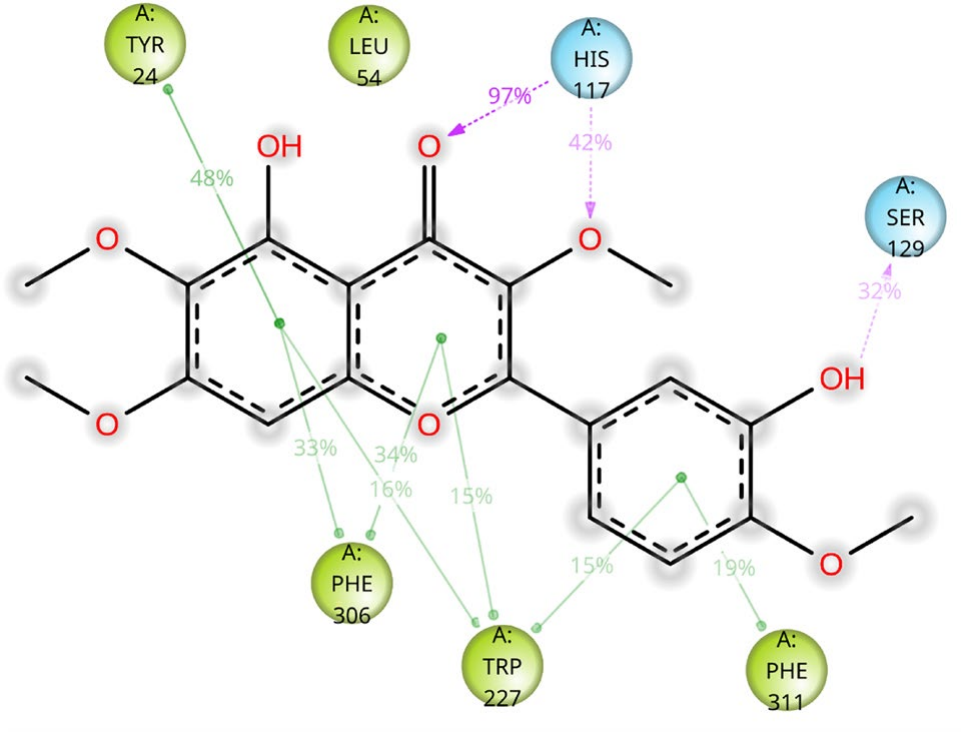
Solid interaction stability throughout the 120 ns MD simulation (replicate #1) of the CAS-AKR1C3 complex, highlighting stable hydrogen bonding and π–π interactions that support the ligand’s inhibitory activity

## Discussion

CAS was evaluated as an inhibitor of AKR1C3, and its inhibitory potential was confirmed using both biochemical and cellular models. In enzymatic assays with recombinant AKR1C3, CAS demonstrated an IC₅₀ value of 5.99 µM, which is within the same order of magnitude as the reference inhibitor ASP9521 (IC₅₀ = 3.32 µM), indicating significant inhibition of the enzyme. Furthermore, CAS suppressed AKR1C3 activity in a cellular context, as evidenced by decreased reduction of coumberone in 22Rv1 cells - an effect comparable to that observed with IND, a known dual COX/AKR1C3 inhibitor. Notably, this dual validation, both at the recombinant enzyme level and in AKR1C3-overexpressing cells, supports the hypothesis that CAS directly targets AKR1C3 in biologically relevant systems.

CAS showed pronounced cytotoxicity in 22Rv1 cells (IC₅₀ = 6.54 µM), whereas its effect on LNCaP cells was markedly weaker (IC₅₀ = 70.90 µM), reflecting an approximately 10-fold difference in potency. Both 22Rv1 and LNCaP are androgen receptor-positive and androgen-dependent cell lines, they differ in key molecular characteristics relevant to therapy resistance. The 22Rv1 cell line is known for high AKR1C3 expression, particularly under castration-level androgen conditions using charcoal-stripped FBS [9, 21]. In contrast, AKR1C3 is undetectable or present only at trace levels in LNCaP cells [7, 9, 22]. Additionally, 22Rv1 expresses splice variants of AR (such as AR-V7), which are specifically associated with resistance to standard treatments [7, 22].

The stark contrast in CAS sensitivity between these two models suggests that its cytotoxic effect is, at least in part, mediated by AKR1C3 inhibition. This interpretation is consistent with the enzyme’s role in sustaining intracrine androgen production and enhancing AR signalling in resistant prostate cancer. Thus, CAS may preferentially affect tumors with elevated AKR1C3 expression, positioning AKR1C3 not only as a pharmacological target but also as a predictive biomarker for therapeutic response. Further studies in additional AKR1C3-positive and -negative models could help validate this selective activity and support stratification strategies in future clinical applications.

Previously, CAS was tested on the androgen-independent DU145 prostate cancer cell line and showed low cytotoxicity (reducing viability to about 80% at 50 µM after 48 h), but also exhibited anti-migratory activity [23]. In other androgen-independent PC-3 prostate cancer cell line CAS was cytotoxic (IC_50_ = 28,8 µM), induced apoptosis and cell cycle arrest at G2/M phase [24]. Furthermore, VAC extracts demonstrated efficacy in inducing cytotoxicity in prostate cancer cells both in vitro and in vivo [25, 26].

CAS was further investigated for its chemosensitizing properties, using ABI and ENZ, standard agents in CRPC therapy, as reference drugs. Concentrations below the IC₅₀ are typically used for synergy evaluation [27]. For each cell line, three sub-IC₅₀ concentrations of CAS were selected. CAS exhibited significant synergistic effects with ABI in 22Rv1 cells. Combined treatment with CAS and ABI in 22Rv1 cells caused a significant reduction (*p* < 0.05) in cell viability compared to treatment with either agent alone.

To quantify the potency of this interaction, the Combination Index (CI) was used. The CI is a quantitative measure to evaluate the interaction between two or more drugs or treatments, indicating whether the combined effect is synergistic (CI < 1), additive (CI = 1), or antagonistic (CI > 1). CI values are typically calculated using the Chou–Talalay method and are widely applied in pharmacology and oncology research to optimize drug combinations [27]. The combination index (CI) analysis demonstrated consistent synergy between CAS and ABI in 22Rv1 prostate cancer cells across the fractional effect range. All tested combinations yielded CI values below 1.0, indicating a synergistic interaction at moderate to high levels of growth inhibition. These findings support the rationale for combinatorial use of CAS to overcome therapeutic resistance in CRPC.

No significant reduction in cell viability was observed in AKR1C3-negative LNCaP cells. Additionally, CAS showed no interaction with enzalutamide (ENZ) in either cell line. Although AKR1C3 inhibitors might be expected to enhance ENZ activity in resistant prostate cancer, the lack of effect could be attributed to off-target effects or other mechanisms, resulting in no overall impact.

In the normal PNT2 prostate epithelial cell line, the combination of CAS and ABI was investigated for its selectivity in combined treatment. While the CAS and ABI combination significantly increased activity across nearly all tested concentrations in the 22Rv1 cancer cell line, a similar effect was observed in normal PNT2 cells in only one instance. Although a trend was noted, statistical analysis suggests that the effect is less pronounced in normal epithelial cells compared to cancer cells.

The enhancement of ABI efficacy through combination with AKR1C3 inhibitors has been previously documented. For example, IND, an AKR1C3 inhibitor, increased ABI efficacy in the resistant C4-2B cell line, and combined treatment significantly reduced tumor volume in SWR22Rv1 mouse xenografts [28]. More recently, a combination of IND and ABI has been investigated in clinical trials [29]. Derivatives of 3-hydroxybenzoisoxazole enhanced ABI’s cytotoxicity against 22Rv1 cells, suppressed PSA expression, and inhibited testosterone production [30]. Likewise, hydroxytriazole analogues not only potentiated ABI activity but also sensitized 22Rv1 cells to ENZ [31].

Analysis of apoptosis in 22Rv1 cells revealed that the drug combination resulted in a statistically significant increase in caspase-3/7 activity compared with either drug used alone. This finding was further supported by Hoechst 33342 staining, which demonstrated an enhanced apoptotic response, evidenced by a higher number of apoptotic bodies in cells treated with the drug combination than with CAS or ABI alone. To date, enhancement of apoptosis has been rarely reported for AKR1C3 inhibitors. Such an effect has been described, for example, for synthetic chromene derivatives [32]; however, there is a lack of evidence for this phenomenon among natural compounds. CAS appears to be the first natural compound shown to potentiate apoptosis when combined with ABI.

Molecular docking positioned CAS within the SP2 hydrophobic pocket, essential for steroid stabilization, where it forms π–π stacking interactions with Trp227 and Phe306. Hydrogen bonds with catalytic tetrad residues Tyr55 (SP2) and His117 (SP3) were also predicted.

During 120-ns molecular dynamics simulations, CAS maintained a hydrogen bond with His117 for 97–98% of the trajectory and remained anchored to at least one aromatic cluster residue (Tyr24, Trp227, Phe306, or Phe311) throughout, each contact occurring ≥ 15% of the time. Particularly stable π–π interactions were observed between the chromen-4-one core and Tyr24 (48%), Trp227 (25%), and Phe306 (43%). Notably, interactions involving the substituted phenol ring, which were not predicted in docking, emerged during MD simulations with Trp227 (28%) and Phe311 (19%), and an additional hydrogen bond with Ser129 persisted for 20–32% of the simulation (for representative interaction profile from simulation replicate #1 see Fig. 10).

In summary, CAS - a flavonoid from VAC - was confirmed as an AKR1C3 inhibitor through recombinant enzyme assays, cellular studies, and molecular modelling. It exhibited potent cytotoxicity against the resistant 22Rv1 line and synergized with ABI. These findings may help explain VAC’s antiandrogenic properties and highlight the therapeutic potential of CAS (and VAC) not only in CRPC but also in other AKR1C3-associated conditions, such as specific cancers, polycystic ovary syndrome, ovarian dysfunction, and hyperandrogenism [10].

## Supplementary Information

Below is the link to the electronic supplementary material.

Supplementary Material 1
