# Supplementary material for: Casticin inhibits AKR1C3 and enhances abiraterone efficacy in castration-resistant prostate cancer
Source: J Nat Med. 2025 Dec 10;80(1):119–29. doi: 10.1007/s11418-025-01974-8 (PMC12847094; doi:10.1007/s11418-025-01974-8)
Supplement: Supplementary file 1 — Supplementary Material 1 [file 11418_2025_1974_MOESM1_ESM.docx]

### Supplementary Information ###


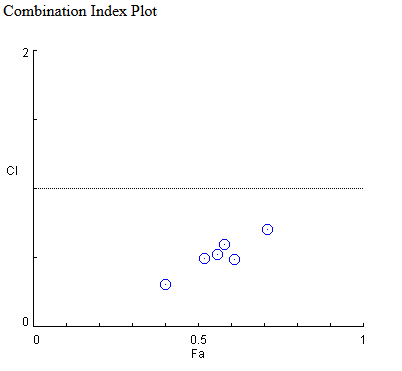


Figure S1. Combination Index (CI) values plotted against fraction affected (Fa) for the combination of casticin and abiraterone in 22Rv1 prostate cancer cells. Data were analyzed using the Chou–Talalay method. All tested combinations yielded CI values below 1.0 in the Fa range of 0.4–0.75, indicating consistent synergistic interaction. CI < 1 denotes synergy, CI = 1 denotes additivity, and CI > 1 indicates antagonism. Data represent the average of at least three independent experiments.

The ligand–protein RMSD plots and interaction timelines from MD simulation replicates #2 and #3.


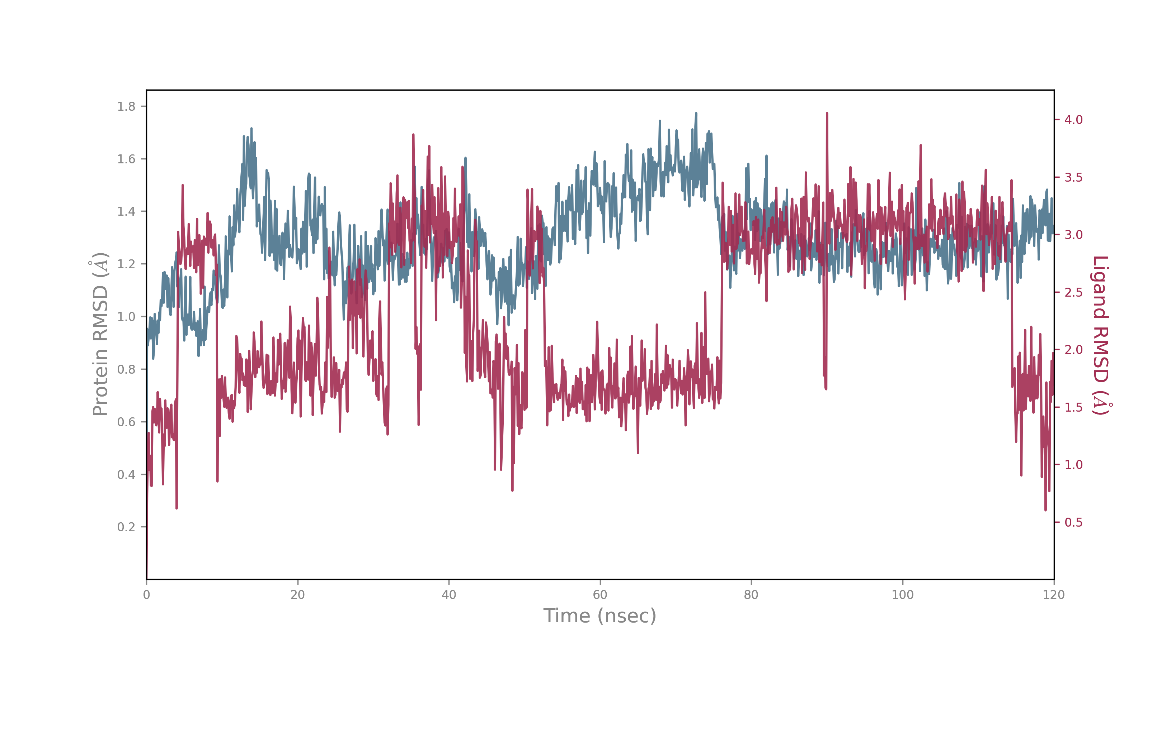


Figure S2. Protein and ligand RMSD values over the 120 ns MD simulation (replicate #2) of the CAS-AKR1C3 complex.


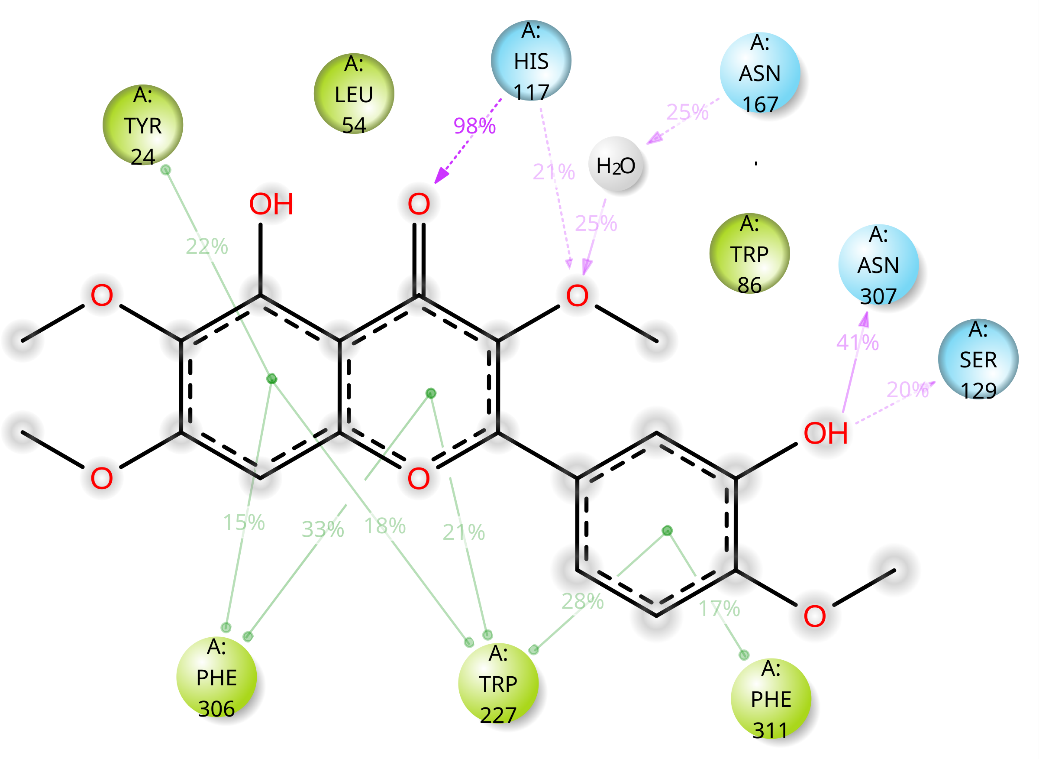


Figure S3. Interaction timeline for the 120 ns MD simulation (replicate #2) of the CAS-AKR1C3 complex.


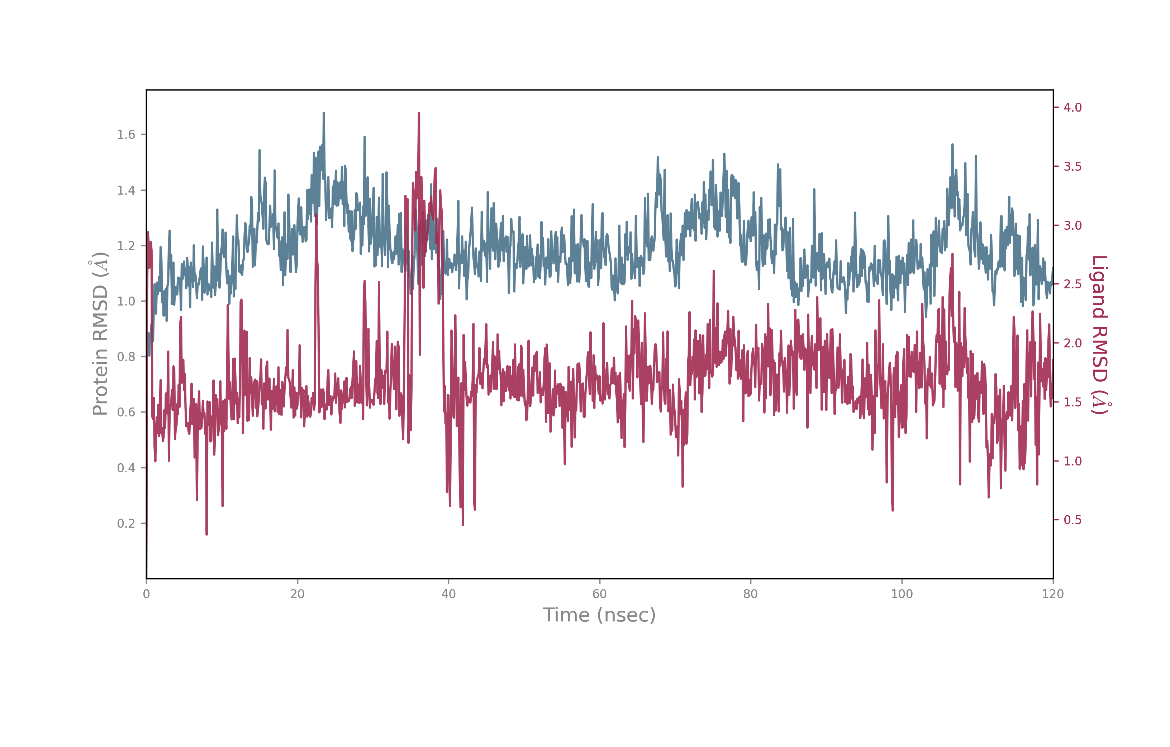


Figure S4. Protein and ligand RMSD values over the 120 ns MD simulation (replicate #3) of the CAS-AKR1C3 complex.


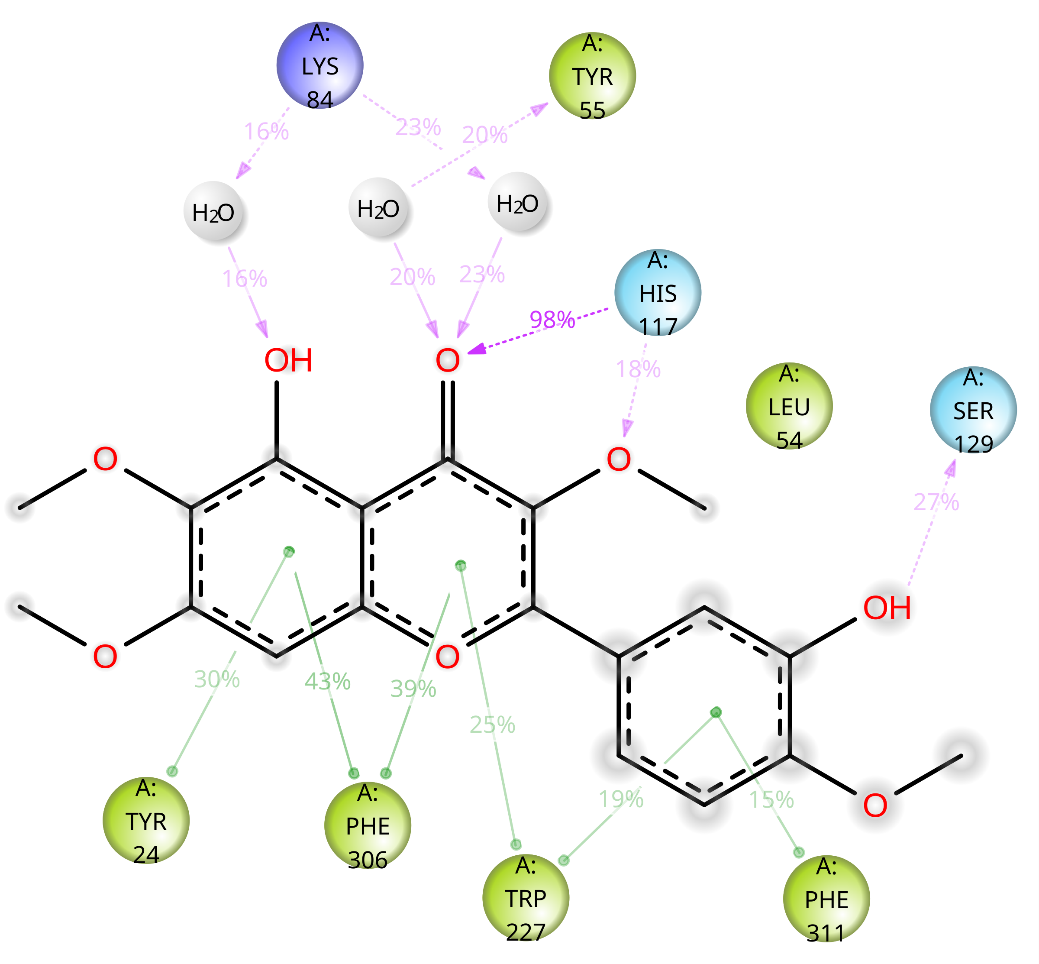


Figure S5. Interaction timeline for the 120 ns MD simulation (replicate #3) of the CAS-AKR1C3 complex.


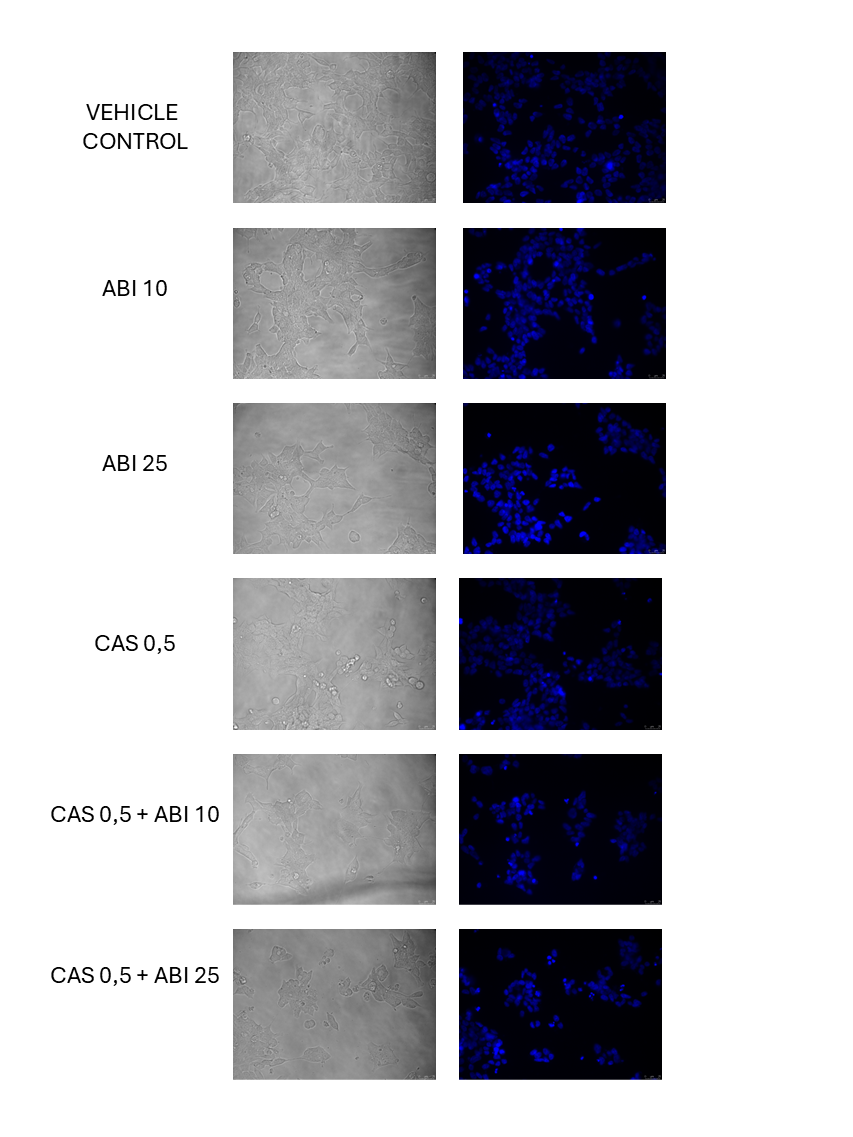


Figure S6. Hoechst 33342 staining of cell nuclei in visible light and fluorescence in 20x magnification.


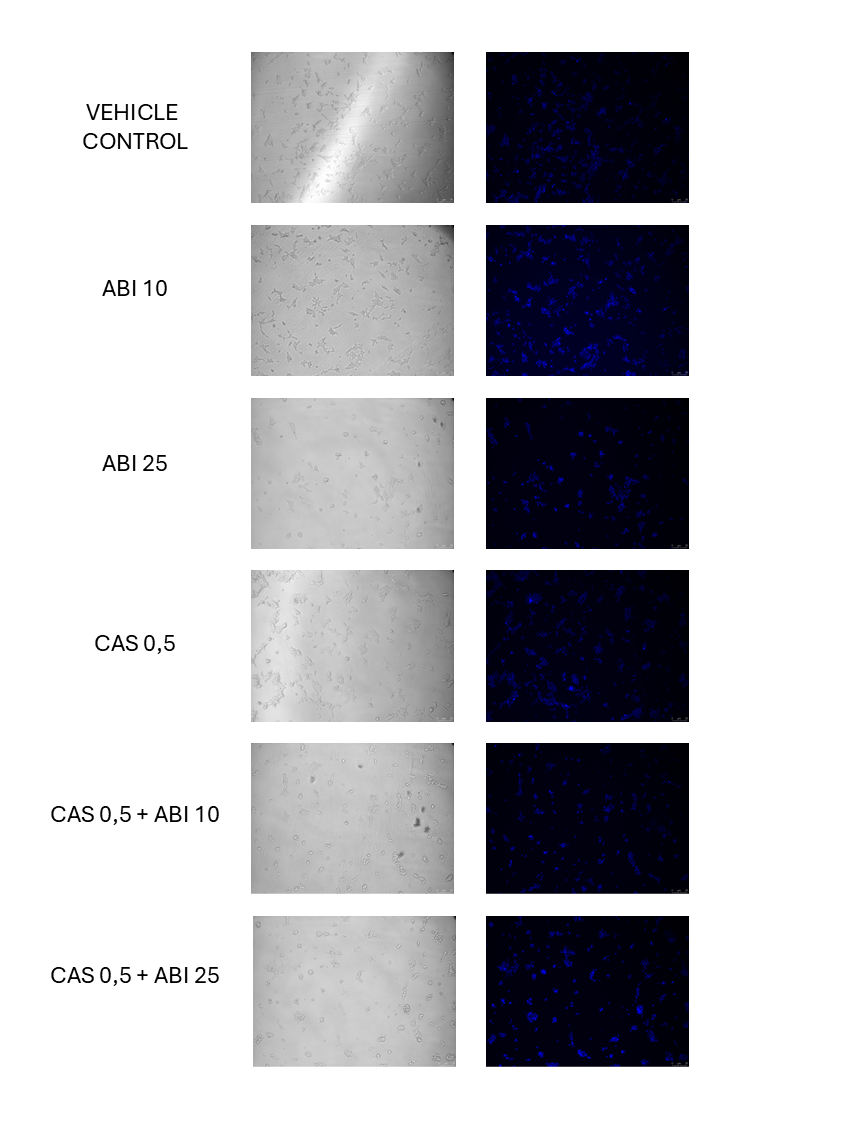


Figure S7. Hoechst 33342 staining of cell nuclei in visible light and fluorescence in 10x magnification.


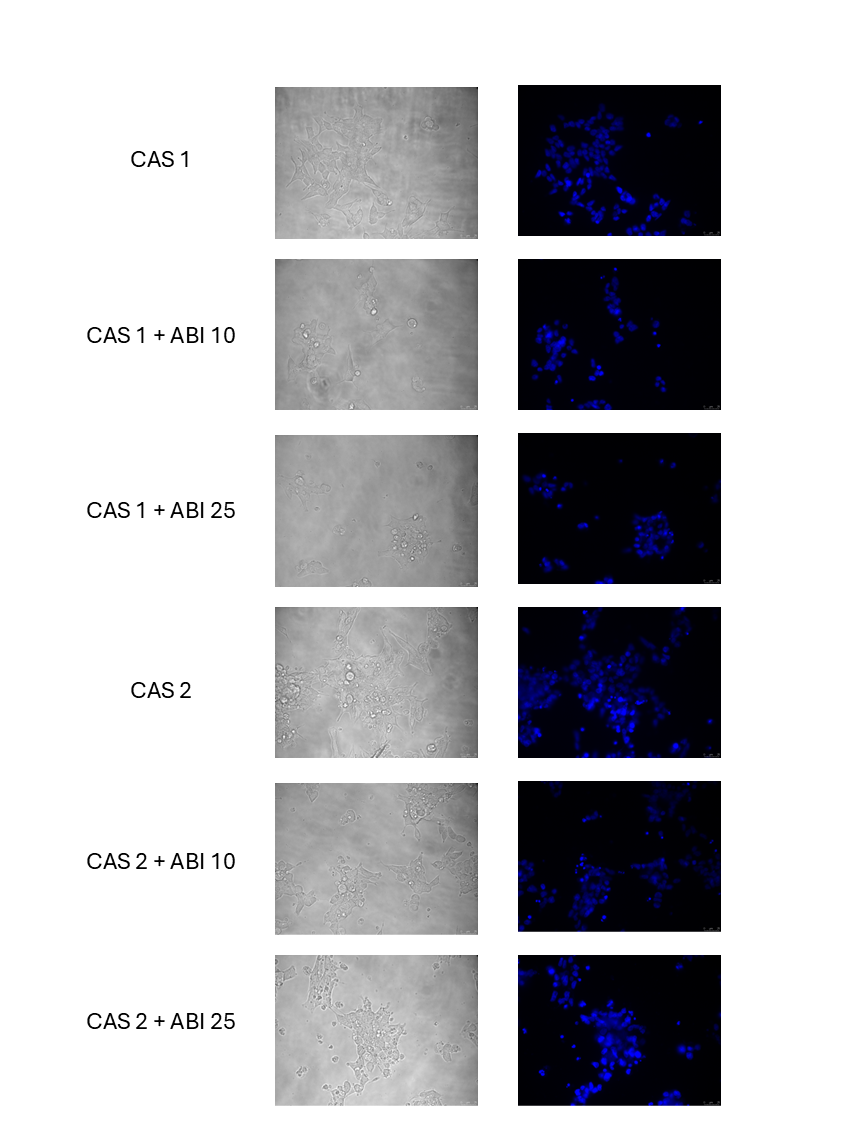


Figure S8. Hoechst 33342 staining of cell nuclei in visible light and fluorescence in 20x magnification.


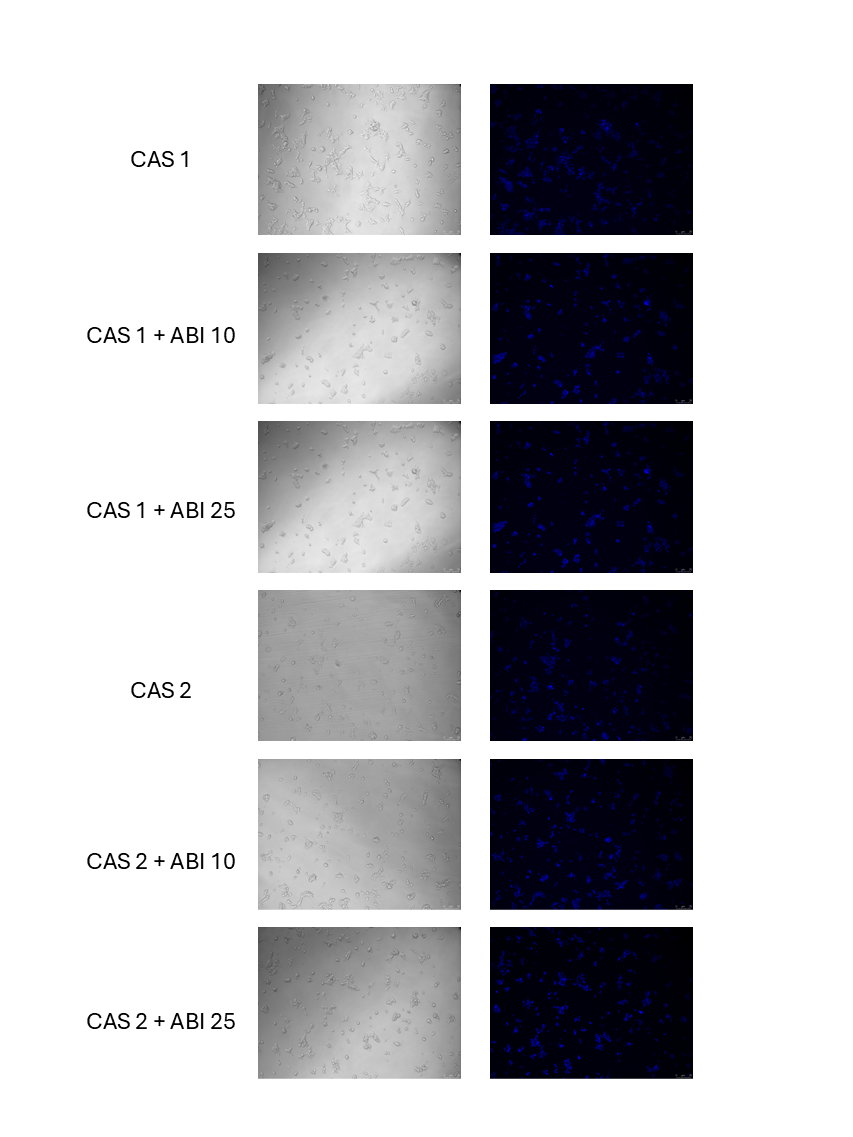


Figure S9. Hoechst 33342 staining of cell nuclei in visible light and fluorescence in 10x magnification.
